# A Case Report of NK-Cell Lymphoproliferative Disease With a Wide Involvement of Digestive Tract Develop Into Epstein–Barr Virus Associated NK/T Cell Lymphoma in an Immunocompetent Patient

**DOI:** 10.1097/MD.0000000000003176

**Published:** 2016-03-25

**Authors:** Haotian Chen, Yu Zhang, Zhinong Jiang, Wei Zhou, Qian Cao

**Affiliations:** From the Department of Gastroenterology (HC, YZ, QC), Department of Pathology (ZJ), Department of General Surgery (WZ), Sir Run Run Shaw Hospital, Zhejiang University School of Medicine (HC, YZ, ZJ, WZ, QC), and Inflammatory Bowel Disease Center of Sir Run Run Shaw Hospital (HC, YZ, ZJ, WZ, QC), Hangzhou, Zhejiang, China.

## Abstract

Epstein–Barr virus (EBV) plays an important role in various diseases. EBV-associated lymphoproliferative disease (LPD) is a rare disease with a canceration tendency. It is difficult to differentiate LPD with involvement of digestive tract from Crohn disease due to similar clinical and endoscopic manifestations. We present a case report of multiple ulcers with esophagus, small bowel and the entire colon involved, proved to be NK-Cell LPD, developed into EBV-associated NK/T Cell lymphoma, in an immunocompetent man who was initially misdiagnosed as Crohn disease.

This report underscores that intestinal ulcers should be cautiously diagnosed, for it sometimes could be a precancerous lesion.

## INTRODUCTION

Epstein–Barr virus (EBV), a linear-DNA herpes virus, plays an etiological role in various diseases, including infectious mononucleosis, chronic active EBV infection, nasopharyngeal cancer, Hodgkin disease and Burkitt lymphoma.^1,2^ EBV-associated lymphoproliferative disease (LPD) is a rare disease and usually observed in individuals with congenital or acquired immune deficiencies. Recently, however, EBV-associated LPD was also reported in immunocompetent individuals.^2–4^ EBV-associated LPD in young adults is a severe chronic active EBV infection with a canceration tendency. It is more prevalent in East Asian countries.^5^ EBV-associated LPD with digestive tract involvement is rather rare and only 5 cases in immunocompetent hosts have been reported so far, 2 cases with B-cell type, 2 cases with T-cell type, and 1 case without cell type reported.^1,2,4,6^ The early diagnosis of LPD will improve the prognosis, but it is challenging for clinicians.

To our knowledge, this is the first reported case of EBV-associated NK-cell LPD with esophagus, small bowel and the entire colon involved developed into an EBV-associated intestinal NK/T-cell lymphoma, misdiagnosed as Crohn disease in an immunocompetent man. This case reminds us that intestinal ulcers, not only Crohn disease, should be diagnosed circumspectly.

### Case Report

A 29-year-old man was admitted to our hospital with recurrent diarrhea and abdominal pain for over 1 year. Four months before the hospitalization, he began to have recurrent irregular fever. Two to 3 days of cephalosporins treatment was effective. Three months later, he went to the local hospital for hospitalization. Gastroscopy and colonoscopy revealed multiple ulcers in esophagus, stomach, terminal ileum, and the entire colon. The histological diagnosis was chronic inflammation. He was diagnosed as Crohn disease and treated with methylprednisolone and mesalazine. The initial dose of methylprednisolone was 45 mg. His symptoms were partly relieved. A week before the hospitalization in our hospital, the abdominal pain reappeared with yellow paste diarrhea, 3 to 4 times daily. Six days later, his abdominal pain intensified accompanied with 3 times of bloody stool, 250 to 300 ml per time. So he was sent to our hospital for anti-TNF treatment.

**FIGURE 1:**
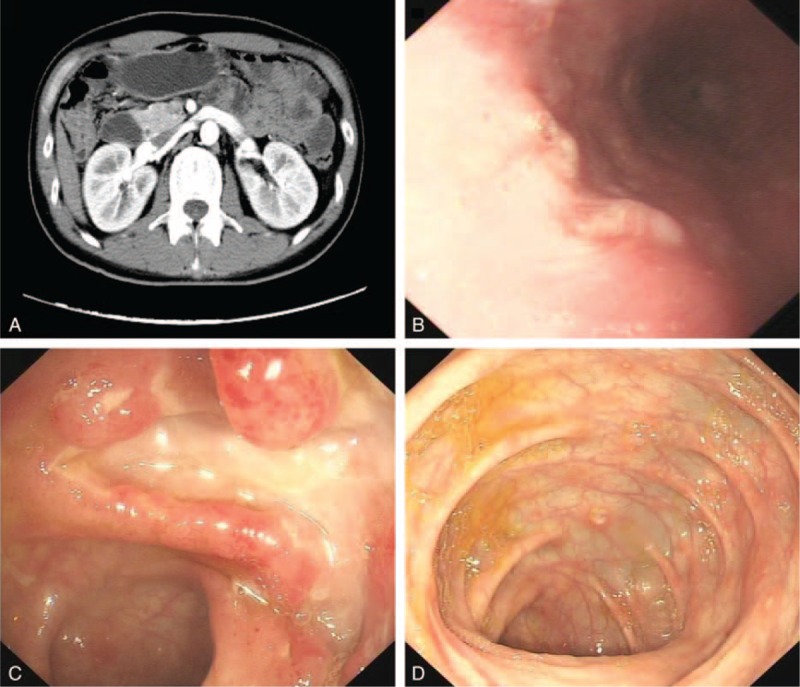
(A) An enhanced small intestine computed tomography shows segmental wall of right colon, ileocecal, and terminal ileum thickened with multiple small lymph nodes seen. (B) Gastroscopy found rough mucosa with a longitudinal ulcer on the lower esophagus. Colonoscopy revealed multiple jumping ulcers in the terminal ileum (C) and the entire colon (D).

**FIGURE 2:**
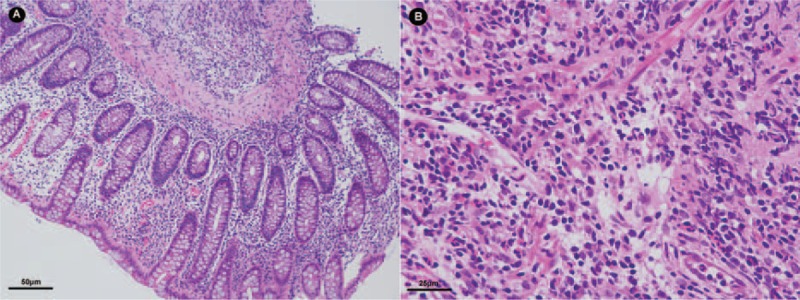
Microscope findings of the ascending colonic mucosa biopsy (A) and esophagus biopsy (200×) (B) in HE stain (400×).

He had no weight loss at presentation. The physical examination revealed mild tenderness on right lower abdomen quadrant. Laboratory tests showed mild leukocytosis (11,700/mm^3^), mild anemia (11.9 g/dL), increased C-reactive protein (CRP) level (37.6 mg/dL), and mild hypoalbuminemia (37.3 g/L). Stool blood was positive. Negative HIV test, negative cytomegalovirus antibody (CMVAb)-IgM and EBVAb-IgM, and a week-positive CMVAb-IgM and EBVAb-IgG were revealed. T-SPOT was negative and no findings in chest computed tomography (CT). The abdominal CT indicated the walls of the right colon, ileocecum, and terminal jejunum thickened (Figure 1A). Gastroscopy found a longitudinal irregular ulcer on the lower esophagus (Figure 1B). Colonoscopy revealed multiple jumping ulcers on the terminal ileum and the entire colon (Figure 1C). Histological examinations of the biopsy samples from the esophagus, terminal ileum, and colon revealed ulcerated mucosa with lymphoid and inflammatory granulation tissue hyperplasia (Figure 2). It seemed that all the evidence pointed to a diagnosis of Crohn disease except his inexplicable high fever, often higher than 39°C since he was admitted. No abscess was found. So we hesitated for his anti-TNF treatment. Bone marrow biopsy gave negative report and no evidence supported a diagnosis of lymphoma.

**FIGURE 3:**
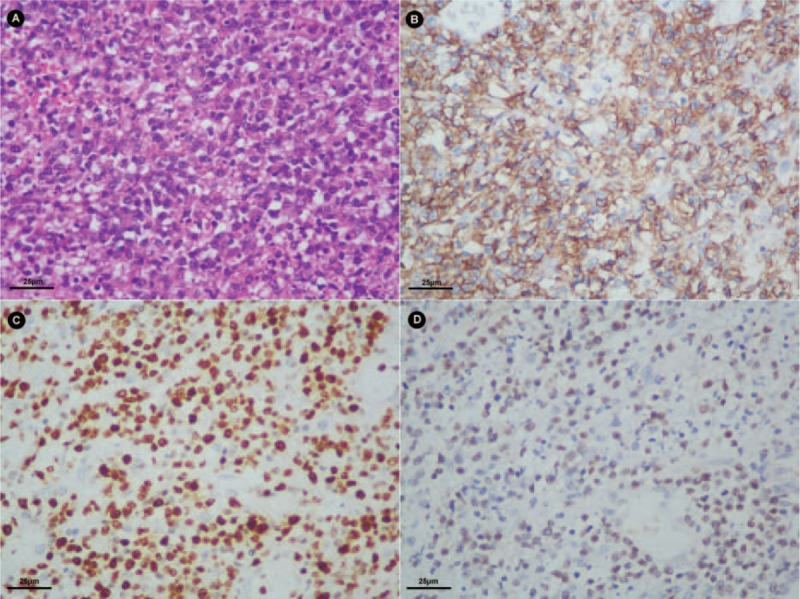
Histologic findings of the lymphoma tissue under the microscope. (A) The cells are large with irregularly shaped or angulated nuclei and a variable amount of cytoplasm (HE stain, 400×). (B) The cells in this case diffusely express CD56 (magnification, 400×). (C) The cells express Ki67 (magnification, 400×). (D) The cells express EBER (magnification, 400×).

**FIGURE 4:**
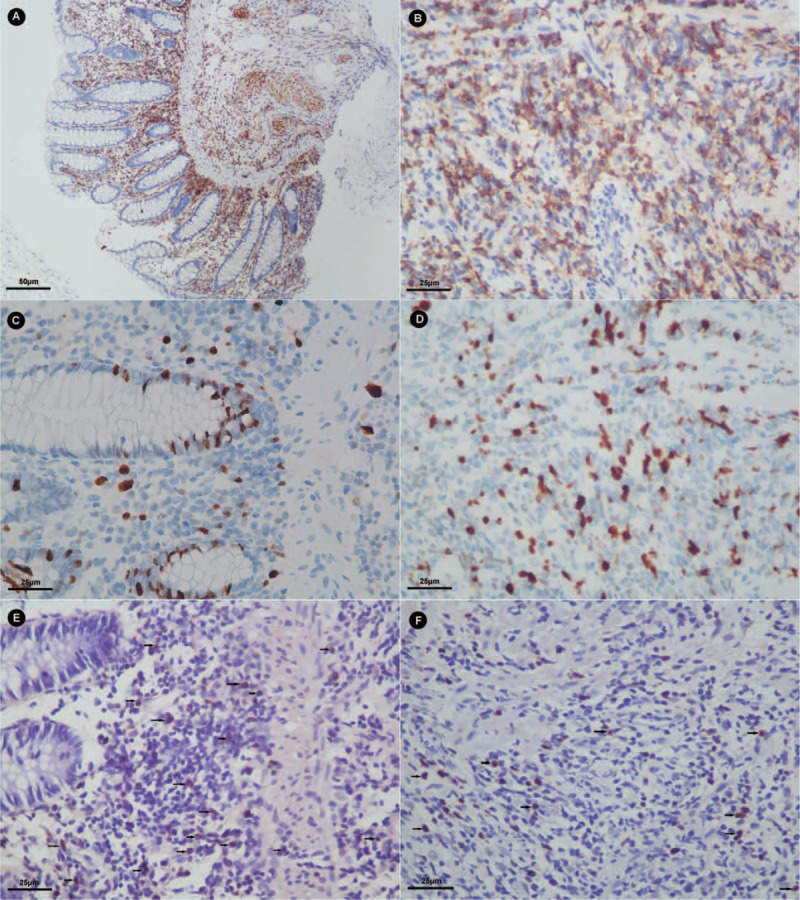
Immunohistochemistry results of CD56 expression (A and B), Ki67 expression (C and D) and EBER expression (E and F) in the ascending colonic mucosa biopsy (A, C, and E) and esophagus biopsy (B, D, and F) (magnification, A in 200× and others in 400×).

After 2 weeks of hospitalization, he developed a fierce abdominal pain. The X-ray prompted an intestinal perforation. An emergency surgery was performed. During the surgery, it was found that there was a 5-cm break in the small bowel 35 cm from the ileocecal valve, the intestinal wall around the break was edematous, and mesenterium was thickened with multiple enlarged lymph nodes. He underwent partial intestinal bowel resection and terminal ileum colostomy. Histological examination of surgical samples revealed lymphoma cells. Immunohistochemical staining (IHC) showed that these cells were diffusely positive for CD56 and EBV-encoded small RNA (EBER) expressions, with a high Ki67 expression rate (Figure 3). He was diagnosed as extranodal NK/T cell lymphoma (nasal type). Then we reviewed the histological slides of the esophagus, small bowel, and the colon biopsies, and we examined the CD56, EBER, and Ki67 expression rates in these samples by IHC. A positive CD56 expression, a positive EBER expression and a 10%-positive Ki67 expression were found in all the esophagus, small bowel, and the colon samples (Figure 4). EBV-associated LPD was made as a pathological diagnosis.

After recovering from surgery, he was started on the CHOP (cyclophosphamide, doxorubicin, vincristine, and prednisolone) and pegaspargase chemotherapy regimen. Two weeks after the first chemotherapy, he suffered a second intestinal perforation. Three weeks later, 1 day after the second chemotherapy, a third perforation happened. The pathological diagnoses were both EBV-associated NK/T-cell LPD with lymphoid hyperplasia in HE stain and positive CD56 and EBER expressions with a 10%-positive Ki67 expression in IHC. The patient died from multiple organ failure 20 days after the last surgery.

## DISCUSSION

EBV is a ubiquitous virus discovered in 1964 and infects over 90% of humans and persists for lifelong.^7,8^ EBV usually infects B cells but also infects T cells and NK cells in rare conditions. EBV nuclear antigen-1 (EBNA1) and EBERs, expressed by EBV-infected cells, has been proposed to cooperate with *c-myc* oncogene and thus contribute to cell proliferation, antiapoptotic, and immune-modulating functions.^9,10^ EBV-associated NK-cell LPD is defined as a systemic illness characterized by proliferation of EBV-infected NK cells.^11^ It is usually observed in patients with congenital or acquired immunodeficiency, while recently it has also been reported in immunocompetent individuals.^2–4^ The so-called immunocompetent is not absolute, they may have some immunodeficiency that we do not know or have not detected yet. Gastrointestinal involvement is rare and the esophagus is the least common site for involvement of lymphoma. This patient has ulcers not only in small bowel and colon, but also in esophagus, and stomach in the first gastroscopy. The ulcers in the stomach disappeared a month later in the second gastroscopy. The histological examination of the first biopsy of gastric mucosa showed a chronic inflammation, with a negative CD56 and positive EBER expression by IHC. We considered that this is an acute EBV-associated gastritis. Similarly, Lavin AC reported a case of multiple ulcerated lesions in the duodenum due to EBV reactivation.^6^ However, the pathological examination reported lymphoid hyperplasia with complete mucosa structure from the biopsy samples obtained from esophagus, terminal ileum, colon and surgical excisions samples from small bowel. The proliferated cells were morphologically normal. The IHC later confirmed a proliferation of NK cells with a low proliferative activity confined to mucous layer, not infiltrated into the submucosa.

Before the first surgery, Crohn disease with upper gastrointestinal tract involved was the most suitable diagnosis for this patient. The jumping ulcers in the colon and terminal ileum, the esophagus ulcer, the thickened intestinal wall in the CT scan, all referred to a diagnosis of Crohn disease. But intestinal ulcer is something more than Crohn disease. Intestinal tuberculosis still remains to be a big problem in developing countries. Lymphoma often lies low and leads to a high misdiagnosis rate. Mansoor et al^12^ proposed a new term “NK-cell enteropathy” to name a disease with multiple NK-cell lymphoproliferative lesions limited to the gastrointestinal tract with undetermined clonality. NK-cell enteropathy, different from EBV-associated LPD, is regarded as a benign disease with a negative EBER expression. Differential diagnosis of intestinal ulcer is truly a big question. Retrospectively, only his inexplicable high fever alerted us to be cautious in this case.

To differentiate LPD with digestive tract involved from Crohn disease is hard but important. EBV-associated LPD should be considered as a differential diagnosis of intestinal ulcers in addition to intestinal tuberculosis and inflammatory bowel disease. As a precancerous condition, the misdiagnosis of EBV-associated LPD would lead to a sad result. Therapy for EBV-associated LPD includes reduction in the dose of immunosuppressive medication. Surgical removal or irradiation of localized lymphoproliferative lesions and interferon-alfa has been effective in some patients.^7,13,14^ Some novel therapeutic approaches such as bone marrow or stem-cell transplantation, even autologous EBV specific T cells therapy has been reported to be effective in some patients.^3,15^ But once the lymphoma come into being, a poor prognosis is often predeterminated.

Extranodal NK/T-cell lymphoma is clinically aggressive and has a poor prognosis. This patient lived for only 3 months since he was diagnosed with EBV-associated NK/T-cell lymphoma. NK/T-cell LPD was first adopted by the WHO as a new category in 2008 and the category was based on pathological features and cell origins.^5^ EBV-infected NK cells proliferate, which indicates that with expanding EBV-infected NK cells, EBV-associated NK-cell LPD might eventually develop into overt leukemia and lymphoma.^11^ Kimura et al^11^ followed 108 patients with EBV-associated T/NK-LPD, 13 patients developed into overt lymphoma or leukemia during a median follow-up period of 46 months. Our patient had a lymphoma in the ileum and EBV-associated LPD lesions in the esophagus, part of the small bowel and the colon. In consideration of his medical course, we proposed that the lymphoma was derived from the LPD lesions although we do not know the exact mechanism.

In conclusion, EBV-associated LPD is a precancerous disease and has a tendency to malignancy. We should be alert when we meet intestinal ulcers for it could be a precancerous lesion rather than a Crohn disease, though it is rather rare. EBV-associated LPD might be the precancerous condition of EBV-associated lymphoma. As we know little about the pathogenic mechanism of EBV in EBV-associated lymphoma, the only way to improve the prognosis is an early detection and early treatment, for the prognosis of LPD would be much better.

## CONSENT

Written informed consent could not be obtained since the patient has deceased and the next of kin were untraceable. We expect that the next of kin would not object to the publication since the patient remains anonymous and this case report gives a worthy clinical lesson.
